# The Effect of Malnutrition on Protein Glycosylation in Children

**Published:** 2014-06

**Authors:** Oznur Bilen, Zekiye Altun, Nur Arslan, Banu Onvural, Pinar Akan, Canan Coker

**Affiliations:** 1 Department of Biochemistry; 2 Oncology Institute; 3 Department of Pediatrics and; 4 Molecular Medicine, Dokuz Eylul University Faculty of Medicine İzmir, Turkey

**Keywords:** Glycosylation; Isoelectric Focusing; Protein Energy Malnutrition; Transferrin; Isoform

## Abstract

***Objective:*** The goal of this study was to evaluate the effect of protein energy malnutrition on protein glycosylation by investigating transferrin isoform pattern and its relationship to the degree of malnutrition and the biochemical markers of nutritional status in children.

***Methods:*** Forty one children with mild (n=23) and severely/moderately (n=18) acute malnutrition and 29 controls were enrolled in the study. Serum transferrin isoforms were determined by isoelectric focusing electrophoresis. Transferrin, prealbumin, zinc, iron and insulin-like growth factor-1 (IGF-1) were measured using automated analyzers.

***Findings*** ***:*** Asialotransferrin and disialotransferrin were significantly higher in severely/moderately malnourished patients compared to controls (*P*=0.04 and *P*=0.04, respectively). Other transferrin isoform patterns were not different among three groups. Serum IGF-1, transferrin and iron levels of severely/ moderately malnourished group were significantly lower than tose of controls (*P*=0.001, 0.02 and 0.03, respectively). Serum prealbumin and zinc levels were similar in all three groups. Serum IGF-1, transferrin and iron levels, and all transferrin isoform patterns were not significantly different in mildly malnutrition group from other two groups.

***Conclusion:*** The changes in transferrin isoform pattern observed in malnourished patients may indicate that malnutrition is a catabolic state which has effects on glycosylation.

## Introduction

Protein glycosylation, a result of many complex reactions in Golgi and endoplasmic reticulum^[^^1^^]^, is closely related to physiological processes such as cell adhesion, migration, cell growth and cell differentiation^[^^2^^]^. Therefore, defects in glycosylation are associated with several pathological conditions including inflammation, rheumatoid arthritis, cancer, liver diseases^[^^3^^]^, galactosemia and fructosemia^[^^4^^]^, sepsis^[^^5^^]^, bacterial meningitis^[^^6^^]^, drugs^[^^7^^]^ and chronic alcohol abuse^[^^8^^,^^9^^]^ in addition to congenital disorders of glycosylation^[^^10^^-^^12^^]^. 

 Protein energy malnutrition is the insufficient and imbalanced consumption of nutrients, characterized by catabolism and consequently, severe morbidity and a high mortality rate^[^^13^^-^^16^^]^. Since malnutrition is considered as a catabolic state, it may influence the glycosylation process and this effect may be related to the severity of malnutrition. 

 To date, *many studies* have been *done* to define the complex *mechanisms* of *glycosylation and to* detect *disorders of glycosylation by analyzing* *different glycoproteins. I*soelectrofocusing of serum transferrin (Tf) is the most widely used screening test for altered glycosylation^[^^11^^,^^12^^]^. Based on its sialic acid (SA) content, human Tf contains at least six different isoforms (glycoforms): pentasialo-, tetrasialo-, trisialo-, disialo-, monosialo-, and asialo-transferrin. The asialo, monosialo and disialo isoforms of transferrin constitute the carbohydrate-deficient transferrin (CDT)^[^^7^^]^. CDT is widely known as a laboratory marker of chronic alcohol abuse for over twenty years. *There *are only *few studies* that investigated the effect of catabolic state on glycosylation. In these studies, CDT has been detected by different methods^[^^17^^-^^19^^]^. The analysis of all Tf isoforms separately was performed in only one of these studies^[^^19^^]^. To our knowledge, there is no study investigating the relationship between protein energy malnutrition and transferrin isoforms in children in the literature.

 The aim of this study was to evaluate the effect of acute malnutrition on glycosylation by investigating the possible changes in transferrin isoform pattern in malnutrition and its relationship to the severity of malnutrition in children.

## Subjects and Methods

**Study population**

This study was conducted in Dokuz Eylül University Faculty of Medicine, Department of Pediatrics, Izmir Turkey. Patients admitted with the complaints of feeding difficulties and/or failure to thrive aged between 6 months and 5 years were enrolled in the study. Height for age and weight for height were calculated for the patients and the severity of acute malnutrition was determined according to Waterlow classification^[^^20^^,^^21^^]^. Sex- and age-matched healthy children admitted to well child outpatient clinic for routine visits constituted the controls. Children were grouped as healthy controls (n=29); mildly (n=23) and severely/ moderately (n=18) [moderately (n=12) and severely (n= 6)] acute malnourished patients. 

 The study protocol was approved by the Ethical Committee of the Dokuz Eylul University Faculty of Medicine and informed consent was obtained from all parents/legal representatives. Chronic hepatic, intestinal, renal, neurologic, metabolic and rheumatologic diseases, acute inflammation and drug usage for any disease are considered as criteria for exclusion. 

**Biochemical analysis**

Venous blood (5 mL) was drawn from a peripheral vein, the samples centrifuged at 1500g for 10 minutes and the sera were frozen at -70˚C until analyzed. Tf and prealbumin concentrations were determined *immunoturbidimetrically* on an auto analyzer (A*rchitect c8000, Abbott Diag., USA*). Zinc and iron levels were measured in serum spectrophotometrically on Architect c8000. Serum insulin-like growth factor-1 (IGF-1) was measured by an automated *chemiluminescence immunoassay* analyzer (I*mmulite* *2500* immunoassay system from Siemens Healthcare Diag, USA).

**Isoelectric focusing (IEF)** **analysis**

Tf isoform analysis was performed by isoelectric focusing (IEF) method^[^^22^^-^^26^^]^. Serum aliquots were incubated at room temperature for 30 min with Fe^III^ solution buffer to saturate the Tf with iron. Isoelectric focusing was performed on Multiphor II electrophoresis unit (Amersham Pharmacia Biotech, Sweden), followed by immunofixation and staining. In short, immobiline dry plate gels, pH 4–7 (GE Healthcare), were rehydrated and then placed on the ceramic cooling plate of the electrofocusing unit (15˚C) and prefocused for 3500 Vh. After *prefocusing, samples* (3 μL of each sample) were applied 1.5 cm away from the cathodic end of the gels. *Tf* isoforms were separated at *high voltages*. *Following IEF, immunofixation was performed using* polyclonal rabbit anti-human Tf antibody (Dako) and subsequently Coomassie Brilliant Blue G-250 (CBB G-250, Amresco) staining was applied as described by Blakesley et al^[^^27^^]^. Densitometric analysis of transferrin isoforms was performed using “Image Master ID Elite Software” (*Amersham*). Ratio of each Tf isoform to *total* *Tf *(%) was determined separately and CDT (sum of *asialo*-, monosialo-, and *disialo*-Tf): *total* Tf *ratio (%)* was also calculated. The imprecision of our method was calculated for disialotransferrin, the *major* constituent of CDT isoforms, and for tetrasialotransferrin, the major glycoform of all transferrin isoforms, using two levels of control material (Recipe). The coefficients of variation (CV) were determined to be <3.5% for within run (n=7) and <7% for between run (n=20) imprecision. 

**Statistical methods**

Data was evaluated using the Statistical Package for Social Sciences (SPSS) 16.0 program for Windows. Data were expressed as mean±standard deviation. Kruskal Wallis test was used for analyzing group averages among three groups. Mann-Whitney U-test was used for comparing two group averages as a post hoc test. Chi-square test was used for comparing group ratios. Intercorrelations between parameters were computed through the Spearman’s correlation analysis. Correlation coefficient indicated low correlation at 0.10–0.29, medium correlation at 0.30–0.49, and high correlation at ≥0.50. All* P-*values were two-tailed and group differences or correlations with *P*<0.05 were considered to be statistically significant.

***Findings***

The mean age and gender were not significantly different among three groups (Table 1). Weight for height of each group was significantly different from that of the other groups. Besides, height for age of both patient groups was lower than that of the control group (Table 1). 

 Serum IGF-1, transferrin and iron levels of severely/moderately malnourished group were significantly lower than those of controls (*P*=0.001, 0.02 and 0.03, respectively) (Table 2). On the other hand, these parameters were not significantly different in mildly malnourished group than in the other groups (Table 2). Serum prealbumin and zinc levels were similar in three groups. 

**Fig. 1:**
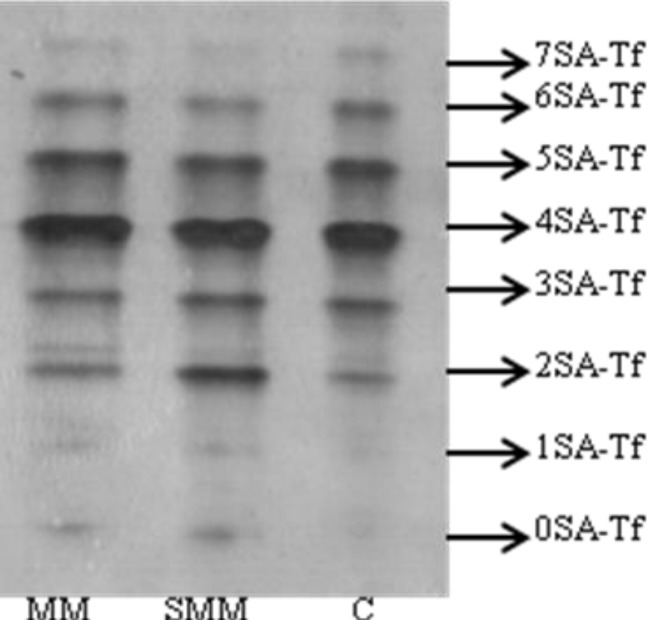
Isoform patterns of Tf in the patients and controls. Numbers (0SA-Tf to 7SA-Tf) on the right indicate different sialotransferrins.

Table 3 and Fig. 1 demonstrate the isoform patterns of Tf in the patients and controls. Significantly higher values were observed for asialotransferrin and disialotransferrin in severely/moderately malnourished group compared to controls (*P*=0.04 and *P*=0.04, respectively). Although monosialotransferrin in severely/moderately malnourished group was higher than in control group, there was no statistically significant difference. Other Tf isoform patterns were not significantly different among three groups. Also, asialotransferrin and disialotransferrin values were not significantly different in mildly malnourished group than in other groups. 

 Weight for height was positively correlated with IGF-1, prealbumin and iron levels in the whole study group. Similarly, iron was positively correlated with prealbumin, IGF-1, zinc, and transferrin levels. Prealbumin was positively correlated with IGF-1 and transferin. Also, IGF-1 was positively correlated with transferin (Table 4).

**Table 1:** Demographic and anthropometric features of patients and controls

**Parameter**	**Severe/moderate** **Malnutrition** **(SMM) (n=18)**	**Mild** **Malnutrition** **(MM) (n=23)**	**Control group ** **(CG) (n=29)**	**Group comparisons ** **(** ***P. *** **value)**
**Age (month)**	15.2 (5.48)	20.78 (11.55)	20.66 (12.64)	SMM-MM (0.2) SMM-CG (0.06) MM-CG (0.4)
**Gender ** **(Female/Male)**	14/4	17/6	15/14	0.06
**Height for age**	88.51 (6.82)	91.56 (5.98)	98.8 (3.55)	SMM-MM (0.08) SMM-CG (<0.001) MM-CG (<0.001)
**Weight for height**	73.47 (4.90)	84.83 (2.85)	97.38 (5.00)	SMM-MM (<0.001) SMM-CG (<0.001) MM-CG (<0.001)

All data presented as mean ± standard deviation and range

**Table 2:** Levels of serum nutritional *markers* and micronutrients in patients and controls (All data presented as mean ± standard deviation and range)

**Parameter**	**Severe/moderate** ** Malnutrition** **(SMM) (n=18)**	**Mild ** **Malnutrition ** **(MM) (n=23)**	**Control group** **(CG) (n=29)**	**Group comparisons** **(** ***P-*** **value)**
**Transferrin ** **(mg/dL)**	243.00 (54.90) (133-375)	275.24 (57.30) (174-378)	275.75 (41.85) (183-382)	SMM-MM (0.09) SMM-CG (0.02) MM-CG (0.6)
**Prealbumin ** **(mg/dL)**	17.66 ( 4.71) (10-28)	17.77 (4.37) (10-31)	19.03 (3.54) (15-26)	SMM-MM (0.7) SMM-CG (0.1) MM-CG (0.3)
**IGF-1 ** **(ng/mL)**	33.17 ( 11.14) (25-62)	44.33 (26.56) (25-126)	57.15 (30.23) (25-130)	SMM-MM (0.4) SMM-CG (0.001) MM-CG (0.07)
**Zinc ** **(μg/dL)**	57.37 (17.61) (35-98)	58.96 (16.44) (26-98)	62.16 (16.12) (34-90)	SMM-MM (0.5) SMM-CG (0.2) MM-CG (0.5)
**Iron ** **(μg/dL)**	41.00 (20.82) (10-79)	48.00 (21.42) (17-97)	63.77 (31.20) (24-121)	SMM-MM (0.5) SMM-CG (0.03) MM-CG (0.1)

IGF-1: Insulin-like growth factor-1

There was a positive correlation between transferrin levels and monosialotransferrin and disialotransferrin in the whole study group. In contrast, transferrin was negatively correlated with tetrasialotransferrin (Table 5). No correlation was detected between iron, IGF-1, prealbumin, zinc and transferrin isoforms (Data not shown).

## Discussion

In this study, the analysis of Tf isoforms by IEF revealed that, serum asialotransferrin and disialotransferrin levels (carbohydrate-deficient transferrins) were significantly increased in severely/moderately malnourished patients in comparison to controls. 

**Table 3:** Transferrin isoforms and carbohydrate-deficient transferrin values of the patients and controls (All data presented as mean ± standard deviation and range)

**Parameter**	**Severe/moderate** **Malnutrition** **(SMM) (n=18)**	**Mild ** **Malnutrition ** **(MM) (n=23)**	**Control group** **(CG) (n=29)**	**Group comparisons** **(** ***P-*** **value)**
**0SA-Tf (%)**	1.76 (1.91) (0.56-8.99)	1.22 (0.70) (0.08-2.59)	1.01 (0.51) (0.04-1.86)	SMM-MM (0.6) SMM-CG (0.04) MM-CG (0.4)
**1SA-Tf (%)**	0.89 (0.78) (0.00-3.30)	0.51 (0.43) (0.02-1.55)	0.62 (0.30) (0.06-1.16)	SMM-MM (0.1) SMM-CG (0.5) MM-CG (0.2)
**2SA-Tf (%)**	5.57 (2.87) (3.53-16)	4.85 (1.58) (2.21-7.89)	4.27 (1.15) (2.28-6.47)	SMM-MM (0.8) SMM-CG (0.04) MM-CG (0.2)
**3SA-Tf (%)**	8.21 (2.22) (3.82-11.40)	8.58 (2.42) (4.10-12.35)	8.86 (2.70) (4.35-13.91)	SMM-MM (0.6) SMM-CG (0.4) MM-CG (0.9)
**4SA-Tf (%)**	57.72 (7.54) (32.81-65.27)	56.38 (5.78) (38.84-64.84)	57.51 (4.84) (45.62-65.95)	SMM-MM (0.8) SMM-CG (0.3) MM-CG (0.5)
**5SA-Tf (%)**	20.97 (2.55) (16.85-24.56)	21.71 (3.49) (16.25-33.39)	20.34 (3.14) (14.20-26.82)	SMM-MM (0.7) SMM-CG (0.4) MM-CG (0.2)
**6SA-Tf (%)**	6.72 (2.42) (3.38-11.74	5.75 (1.98) (3.27-11.70)	6.29 (2.04) (1.92-12.36)	SMM-MM (0.2) SMM-CG (0.7) MM-CG (0.3)
**7SA-Tf (%)**	1.11 (0.75) (0.04-3.31)	0.82 (0.65) (0.00-2.90)	1.04 (0.57) (0.16-2.47)	SMM-MM (0.2) SMM-CG (0.7) MM-CG (0.2)

SA: sialic acid; TF: transferrin

**Table 4:** Correlation between anthropometric features and nutritional laboratory parameters in patients and controls (n=70)

	**weight for height**	**Iron**	**Prealbumin**	**IGF-1**	**Zinc**
**Iron**	0.29*				
**Prealbumin**	0.25*	0.35‡			
**IGF-1**	0.39‡	0.59‡	0.45‡		
**Zinc**	0.24	0.29*	0.20	-0.08	
**Transferrin**	0.24	0.27*	0.25*	0.46‡	0.10

IGF-1: Insulin-like growth factor-1

* Correlation is significant at the 0.05 level;

‡ Correlation is significant at the 0.01 level

Our results confirmed that CDT isoforms increase in malnutrition and these findings *support the opinion that a* relationship exists between catabolic diseases and protein glycosylation. To our knowledge, this is the first study in the literature investigating the relationship between protein energy malnutrition and transferrin isoforms in children.

 CDT was reported to be a promising marker for assessment *of nutritional status* in catabolic patients in different studies. All of these studies showed that low body mass index correlated with higher CDT levels^[^^17^^,^^28^^-^^31^^]^ or high body mass index was associated with lower CDT levels^[^^32^^]^. There appears to be an inverse relationship between* catabolic states due to psychiatric disorders (in *63% of the patients*) distinct from alcoholism*^[^^17^^]^.

 Glycosyltransferases, enzymes which are involved in the glycosylation of Tf, are inhibited by the ethanol metabolite acetaldehyde. It is suggested that the biochemical mechanism underlying the CDT elevation in catabolic patients may be the *structural similarities between acetoacetate* and acetaldehyde. Therefore, it is possible that acetoacetate could also inhibit glycosyl-transferases resulting in an increase of CDT. 

 Ano*ther* *study* by *Reif et al* showed that anorexia nervosa (AN) patients had elevated CDT values in 57% of cases; on the other hand, bulimia nervosa patients had normal CDT levels^[^^18^^]^. The body mass index and CDT levels^[^^7^^]^. *Reif et al have shown that CDT levels increased significantly in *difference between CDT levels in the two patient groups was explained by the fact that catabolic metabolism is more marked in AN.

MM: A mildly malnourished patient, SMM: A severely/ moderately malnourished patient, C: Control

*Furthermore*, patients with initially elevated CDT tended to be more seriously ill than those without. Another issue is that during therapy, as the body mass index of anorexia nervosa patients increased to normal levels, CDT inversely declined^[^^18^^]^. In a recent study by Arndt et al, CDT was analyzed in AN patients by three different methods including high performance liquid chromatography (HPLC), capillary electrophoresis (CE) and immunoassay. However, in contrast to the immunoassay tests, no elevation of CDT in anorexia nervosa patients was detected by HPLC and CE^[^^19^^]^. An interesting finding of this study was increased trisialotransferrin isoform that was detected by HPLC in 67% of AN patients.

**Table 5:** Correlation between serum transferrin levels and transferrin isoforms

	**0SA-Tf **	**1SA-Tf**	**2SA-Tf**	**3SA-Tf**	**4SA-Tf**	**5SA-Tf**	**6SA-Tf**	**7SA-Tf**
**Transferrin (n=70)**	0.16	0.28*	0.33‡	0.18	-0.31‡	-0.11	0.32*	0.03

* Correlation is significant at the 0.05 level/

‡ Correlation is significant at the 0.01 level

Thus, it is suggested that elevated CDT values from the past studies using immunoassay method are most likely due to an incomplete separation of trisialotransferrin from CDT on the previous fractionation step and thus overdetermination of CDT. On the other hand, these results indicate that trisialotransferrin fraction may be used as a laboratory marker of (hypocaloric) malnutrition in this study^[^^19^^]^. 

 In our study, transferrin was the only laboratory parameter showing correlation with transferrin isoforms. It was positively correlated with monosialotransferrin, disialotransferrin (CDT group), and hexasialotransferrin while negatively correlated with tetrasialotransferrin. It seems that the latter condition can be relevant to analytical process rather than the clinical status. Tetrasialotransferrin overload occurs when adequate sera for detecting all isoforms were applied to gel medium. Consequently, the intensity of tetrasialotransferrin bands would not be correlated with tetrasialotransferrin concentration. Relatively low tetrasialotransferrin isoform in higher transferrin concentrations has been explained by this analytical condition. The effect of serum iron and transferrin levels on CDT is not clear. Low iron and increased transferrin levels can increase total levels of CDT and possibly %CDT levels^[^^7^^]^. In our study, although both iron and transferrin levels decreased in the severely/moderately malnourished groups, asialotransferrin and disialotransferrin increased in these patients. Moreover, in our study, there was no correlation between iron and transferrin isoforms. So, it can be concluded that transferin levels can affect the di- and hexasialotransferrin levels in these groups. Brathen et al found in their study that both low and high serum transferrin levels could be associated with elevated CDT levels^[^^28^^]^. In the literature, three adult studies showed inverse relationship between iron and CDT levels^[^^31^^,^^33^^,^^34^^]^, whereas Stauber et al found no relationship between iron levels and CDT values^[^^35^^]^. There is insufficient data to draw any conclusions about the effect of iron and transferrin on CDT values; for this reason, clinicians should analyze the elevated CDT results carefully in the patients with low iron levels, particularly in malnourished patients. 

 One major *limitation* of our *study* is the low *number *of severely (n=6) malnourished patients. In a larger patient group with severe malnutrition and in other disease states with markedly increased catabolism, transferrin isoform analysis with isoelectric focusing may reveal the alterations in protein glycosylation. Another limitation of this study is the case-control design of the study. Prospective studies, which analyze the alterations of transferrin isoforms during therapy and investigate the relationship between the weight gain and tranferrin isoforms are needed. 

## Conclusion

The results of this study point out that malnutrition may affect glycosylation in children. The mechanisms underlying the alterations in glycosylation during catabolic process and its outcomes need to be elucidated with further clinical and laboratory studies.

## Authors Contribution

O. Bilen and N. Arslan have primary responsibility for protocol development, patient screening, enrollment, laboratory experiments, data analysis and writing the manuscript. 

Z. Altun and P. Akan participated in the development of the protocol and analytic framework for the study. 

B. Onvural and C. Çoker supervised the design of the study, performed the final data analyses. 

All Authors approved the final version of the paper.
